# Gas sensing performance of In_2_O_3_ nanostructures: A mini review

**DOI:** 10.3389/fchem.2023.1174207

**Published:** 2023-04-07

**Authors:** Shulin Yang, Huan Yin, Zhao Wang, Gui Lei, Huoxi Xu, Zhigao Lan, Haoshuang Gu

**Affiliations:** ^1^ Hubei Key Laboratory for Processing and Application of Catalytic Materials, School of Physics and Electronic Information, Huanggang Normal University, Huanggang, China; ^2^ Hubei Key Laboratory of Ferro and Piezoelectric Materials and Devices, Faculty of Physics and Electronic Sciences, Hubei University, Wuhan, China

**Keywords:** In_2_O_3_, nanostructrues, gas sensor, sensing mechanism, review

## Abstract

Effective detection of toxic and hazardous gases is crucial for ensuring human safety, and high-performance metal oxide-based gas sensors play an important role in achieving this goal. In_2_O_3_ is a widely used n-type metal oxide in gas sensors, and various In_2_O_3_ nanostructures have been synthesized for detecting small gas molecules. In this review, we provide a brief summary of current research on In_2_O_3_-based gas sensors. We discuss methods for synthesizing In_2_O_3_ nanostructures with various morphologies, and mainly review the sensing behaviors of these structures in order to better understand their potential in gas sensors. Additionally, the sensing mechanism of In_2_O_3_ nanostructures is discussed. Our review further indicates that In_2_O_3_-based nanomaterials hold great promise for assembling high-performance gas sensors.

## 1 Introduction

In recent decades, there has been growing attention to the effective monitoring of air quality due to the increasingly serious environmental problems (Ma et al., 2016; Zhang et al., 2018; Ge et al., 2019). Even toxic gases with low concentrations can be harmful to human health (Park et al., 2016; Cordero et al., 2018; Zhou et al., 2022). For example, the toxic gas formaldehyde (HCHO) can cause serious blurred vision and vertigo when its concentration exceeds 0.1 mg/m^3^ (Peng and Huang, 2022). In the workplace, the concentration of n-butanol should be kept below 152 mg/m^3^ to ensure the safety of human lives (Zhao et al., 2021). In addition, a high risk of explosion may occur if the concentration of H_2_ reaches 4%–75% in the air (Phanichphant, 2014). It should be noted that many toxic, hazardous, or flammable gases are odorless, colorless, and tasteless, which means they cannot be detected by humans directly (Wetchakun et al., 2011; Chi et al., 2014; Shi et al., 2018). Therefore, high-performance gas sensors are of great importance to effectively detect these gases and their concentrations in the air.

Metal oxide-based gas sensors have become a popular research topic in recent years due to their advantages of low cost, easy of fabrication, low power consumption and high sensor response to a wide range of gases (Xu and Cheng, 2016; Lu et al., 2019; Nikolic et al., 2020). Nanostructured metal oxides always presented high specific surface areas and could provide abundant active sites on their surfaces (Zhang et al., 2017; Srinivasan et al., 2019; Walker et al., 2019). This positive factor effectively promotes the adsorption and the diffusion of gas molecules in the sensing materials, resulting in the excellent gas sensing performances of nanostructured metal oxides. For instance, in the study conducted by Zhu et al., CuO nanoflowers demonstrated a significantly higher sensor response of 123.4 to 50 ppm H_2_S at 80°C compared to CuO-based microspheres, which only showed a sensor response of 4.36 (Hu et al., 2017; Hu et al., 2018a). Chen et al. also reported superior sensing performance of ZnO-based nanostructures with a sensor response as high as 6043 to 100 ppm triethylamine (TEA) at an optimal working temperature of 183.5°C, when compared to ZnO films (with a response of ∼22.5) or hierarchical ZnO microspheres (with a response of 242) (Shen et al., 2018; Liu et al., 2021a; Li et al., 2021). Furthermore, the net-like SnO_2_ nanoarrays showed a response time of only 16.3 s to 10 ppm H_2_S at 350°C, which was approximately ten times lower than that of SnO_2_ films (167.8 s) (Ge et al., 2022). Thus, outstanding gas sensing properties could be expected through synthesizing nanostructured metal oxides.

In_2_O_3_ is another popular n-type metal oxide that possesses a wide band gap of 3.5–3.7 eV (Vuong et al., 2014; Han et al., 2015; Park, 2017). Its outstanding thermal stability, high conductivity, and excellent chemical/physical properties make it a promising candidate for gas detection (Liang et al., 2015; Kumar et al., 2021; Meng et al., 2022). For example, Zhang et al. successfully prepared Ni-doped In_2_O_3_-based nanocubes through a hydrothermal method, achieving effective detection of 20 ppm HCHO with a response time of 76 s at room temperature (Zhang et al., 2020). The research conducted by Han et al. demonstrated that the sensor response of In_2_O_3_ nanorods doped with Co could be improved to 23.2 towards 10 ppm HCHO at 130 C (Wang et al., 2018). Additionally, flower-like In_2_O_3_ nanomaterials exhibited a sensor response and response time of 3.1 and 53 s, respectively, to 0.5 ppm isoprene at 190°C (Han et al., 2020). A Google Scholar survey with keywords of “nano + In_2_O_3_+gas sensor” revealed that from 2017 to 2022, there were 826, 878, 919, 1060, 1210, and 1520 papers published on the topic. Although the data obtained may not be entirely accurate, the increasing number of published references highlights the growing attention given to In_2_O_3_-based gas sensors in recent years. Therefore, summarizing the recent developments in In_2_O_3_-based gas sensors would be meaningful to better understand their advantages in gas sensing.

In this paper, we have chosen several highly cited published references to conduct a mini review on typical In_2_O_3_-based gas sensors. Our focus was mainly on summarizing and comparing the high-performance characteristics of these gas sensors. Furthermore, we presented the methods used to prepare various In_2_O_3_-based materials. Additionally, we provided a brief review of the gas sensing mechanism for In_2_O_3_-based gas sensors.

## 2 Research status of gas sensing performances of recent In_2_O_3_ nanostructures

### 2.1 Pristine In_2_O_3_-based nanomaterials

**FIGURE 1:**
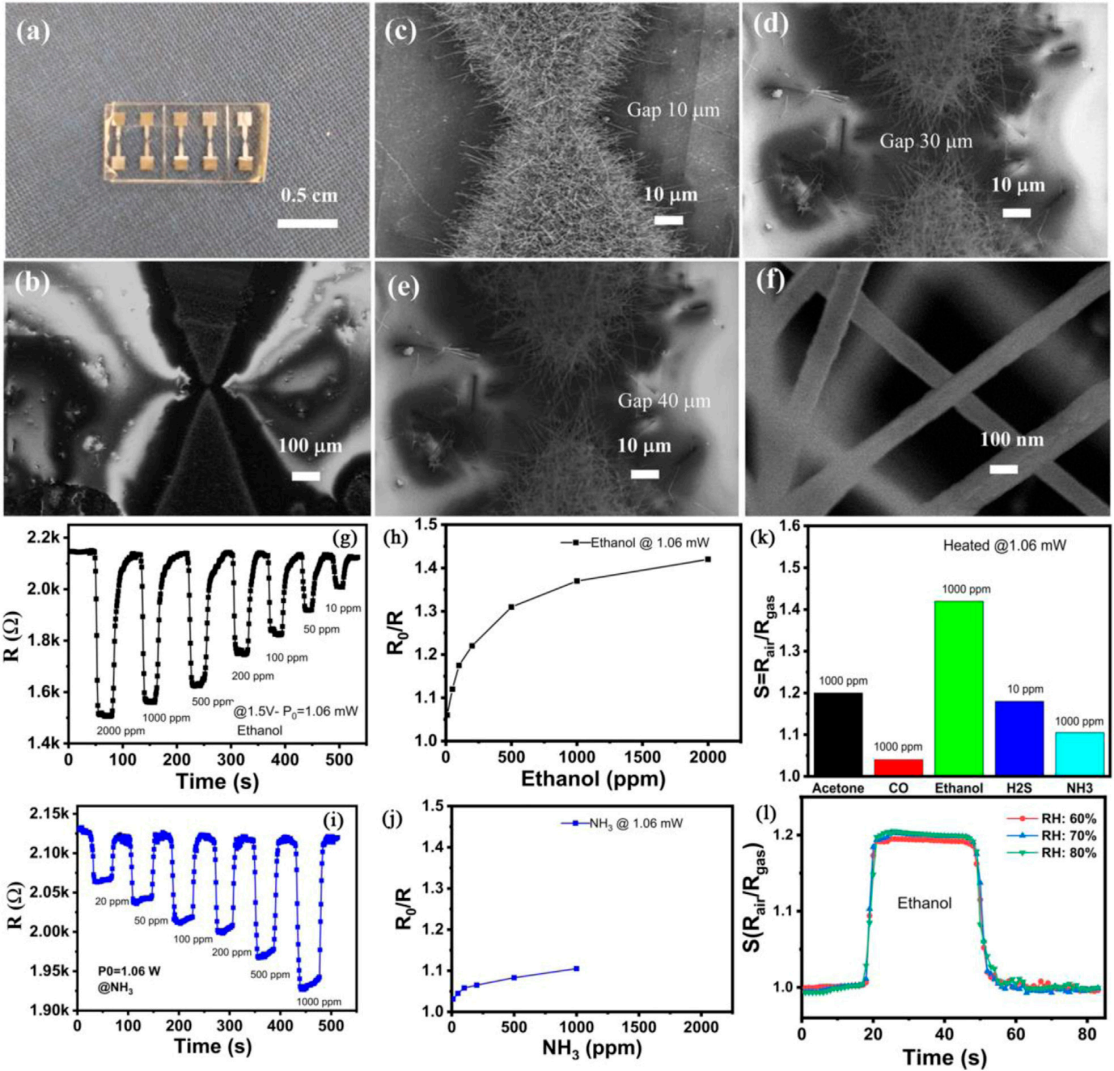
**(A)** Digital image of the assembled gas sensor. **(B)** SEM image of a gas sensor. **(C–E)** SEM image of In_2_O_3_ nanowires grown at electrodes with gasps of 10, 30 and 40 μm, respectively. **(F)** High magnification SEM of In_2_O_3_ nanowires. **(G, H)** Dynamic gas sensing performance and sensor response **(I, J)** of sensor-40 to 10–2000 ppm ethanol or NH_3_ at room temperature. **(K)** Selective gas sensing performance of sensor-40. **(L)** ethanol gas sensing behavior of sensor-40 under different humidity. Reprinted with permission from ref. (Son et al., 2022). Copyright 2022, Elsevier.

A novel self-heated gas sensor for detecting ethanol at room temperature was assembled by Nguyen et al. using In_2_O_3_ nanowires (Son et al., 2022). The sensor utilized the Joule effect generated by the In_2_O_3_ nanowires under an operating voltage to achieve self-heating during operation. The In_2_O_3_ nanowires were synthesized *via* a one-chip growth technique of thermal evaporation. The gap between the prepared electrodes was designed to be 10, 30 or 40 µm (Figures 1A, B), with the corresponding devices labeled as sensor-10, sensor-30, or sensor-40, respectively. Results showed that the well-crystallized In_2_O_3_ nanowires were successful to bridge the gap of electrodes (Figures 1C–E). The In_2_O_3_ nanowires had an average diameter of ∼100 nm and an average length of over 10 µm (Figure 1F). Sensor-40 exhibited better ethanol sensing performance compared to sensor-10 or sensor-30. Sensor-40 showed a superior sensing performance to 10–2000 ppm ethanol compared to NH_3_ under a supplied power of 1.06 mW (Figures 1G–J). The sensor response of sensor-40–2000 ppm ethanol was ∼1.45. Meanwhile, the sensor response of sensor-40–1000 ppm ethanol was higher than that to 1000 ppm acetone, CO, H_2_S or NH_3_ (Figure 1K), indicating good gas selectivity of the In_2_O_3_ nanowires. Additionally, the sensor response of sensor-40 to ethanol was not significantly affected by humidity levels of 60%, 70%, or 80% (Figure 1L).

Shboul et al. have developed a novel gas sensor mainly composed of solution-printed In2O3 nanoparticles (Al Shboul and Izquierdo, 2021). In their study, In_2_O_3_ nanoparticles, copper acetate (CuAc), graphite (Gt) flakes, and polystyrene (PS) were added to 10 mL of xylene to form a stable paste. The paste was then spread over a flexible PET substrate with prepared carbon electrodes to assemble the gas sensor. The In_2_O_3_-based sensor (SS), without CuAc, showed unresponsive sensing performance to H_2_S showed unresponsive sensing behavior towards H_2_S with concentrations below 100 ppb. The sensor response of the SS was only 2–100 ppb H_2_S, and the response time was as long as ∼25 min. The In_2_O_3_-based sensing material with 10 wt% CuAc (MS10) exhibited better sensing performance than sensing materials with 2 wt% CuAc (MS2), 25 wt% CuAc (MS25), or 50 wt% CuAc (MS50). The sensor response of the MS10 was found to be ∼18–100 ppm H_2_S at room temperature, with a relative humidity of ∼30%.

The study by Pham et al. also showed the potential of porous In_2_O_3_ nanorods for detecting CO gas at 350°C (Van Tong et al., 2021). The nanorods were synthesized *via* a hydrothermal method at 180°C for 10 h, and showed a sensor response of 3.46–400 ppm CO with a response and recovery time of 41/43 s. Similarly, Zhang et al. employed a surfactant-assisted co-precipitation method to prepare hierarchical branch-like In_2_O_3_ nanomaterial for detecting ozone (O_3_) (Sui et al., 2021). The resulting hierarchical branch-like In_2_O_3_ showed a high sensor response of 44–100 ppb O_3_ at its optimum working temperature of 70°C.

### 2.2 In_2_O_3_-based composites

#### 2.2.1 In_2_O_3_ composited with noble metals

Wang et al. have investigated the impacts of Au, Ag, Pt, and Pd on the ethanol gas sensing performance of long-range mesoporous In_2_O_3_ (Cheng et al., 2021). They synthesized the ordered mesoporous In_2_O_3_ by replicating the structure of SBA-15, and then prepared Au, Ag, Pt, or Pd-doped In_2_O_3_ through an *in-situ* doping routine. The mesoporous In_2_O_3_ doped with Pd exhibited a specific surface area of 94.22 m^2^/g, significantly higher than that of pristine In_2_O_3_ (64.55 m^2^/g), Au-doped In_2_O_3_ (78.29 m^2^/g), Ag-doped In_2_O_3_ (67.52 m^2^/g), or Pt-doped In_2_O_3_ (76.41 m^2^/g). Similarly, the pore diameter for the Pd-doped In_2_O_3_ was 3.6 nm, also larger than that for pristine In_2_O_3_ (2.6 nm). The high specific surface area and large pore diameter are favorable for improving gas molecule adsorption and diffusion in the sensing material. Consequently, the concentration of chemisorbed oxygen species in Pd-doped In_2_O_3_ was the highest among all samples, reaching 54.8%. The sensor based on Pd-doped In_2_O_3_ also demonstrated the best performance for 100 ppm ethanol at an operating temperature of 200–350°C. The sensor response of Pd-doped In_2_O_3_ was 39 at 250°C, higher than that of pristine In_2_O_3_ (∼5), Au-doped In_2_O_3_ (∼7.5), Ag-doped In_2_O_3_ (∼15), or Pt-doped In_2_O_3_ (∼17.5). Furthermore, Pd-doped In_2_O_3_ exhibited higher sensor response to 100 ppm ethanol than to 100 ppm ammonia, methanol, toluene, benzene, acetone, formaldehyde, or ethanol, revealing excellent sensing selectivity.

The research conducted by Zhang et al. showed that an excellent hydrogen sensing performance could be achieved with the use of Tb-doped In_2_O_3_ nanocomposites decorated with Ag (Ag-Tb-In_2_O_3_) (Bai et al., 2022). In their study, the Ag-modified Tb-doped In_2_O_3_ nanocomposite was successfully synthesized through a hydrothermal process combined with a facile annealing method. Interestingly, the material exhibited two coexisting crystalline phases, including the hexagonal phase In_2_O_3_ (h-In_2_O_3_) and the cubic phase In_2_O_3_ (c-In_2_O_3_). Further analysis of the XRD patterns confirmed that the Tb was doped within the In_2_O_3_ while the Ag was decorated on the surface of the nanocomposite. The Tb doping was found to reduce the grain sizes of both c-In_2_O_3_ and h-In_2_O_3_, resulting in the generation of oxygen vacancies in the nanocomposite. Consequently, the Ag-Tb-In_2_O_3_ nanocomposite exhibited a better sensing performance for 500 ppb H_2_ at operating temperatures ranging from 120 to 200°C. The sensor response of the Ag-Tb-In_2_O_3_ was found to be 4.63–500 ppb H_2_ at its optimum operating temperature of 160°C, which is higher than that of the pristine In_2_O_3_ (∼1.5), Tb-doped In_2_O_3_ (∼2.5), or Ag-decorated In_2_O_3_ (∼3.5).

#### 2.2.2 In_2_O_3_ composited with metal oxides

The study by Xie et al. demonstrated that the hydrogen sensing performance of In_2_O_3_ nanotubes could be significantly enhanced by co-doping them with PdO and NiO (Luo et al., 2021). Pristine In_2_O_3_ and In_2_O_3_ doped with NiO, PdO or NiO/PdO were synthesized using an electrospinning method. All four samples exhibited sensing performances to 5 ppm hydrogen gas at 160–300°C. Among them, the PdO/NiO-In_2_O_3_ nanotubes showed the highest sensor response, with a value of 487.52 to 5 ppm H_2_ at 160°C. In contrast, the sensor responses of pristine In_2_O_3_, NiO-In_2_O_3_, and PdO-In_2_O_3_ were lower than 20. The response times of the pristine In_2_O_3_ and NiO-In_2_O_3_ were also relatively long, at 153 s and 97 s, respectively, which might not be suitable for rapid detection of hydrogen gas in practical applications. However, the addition of PdO significantly reduced the response time to only 1 s for both PdO-In_2_O_3_ and PdO/NiO-In_2_O_3_, demonstrating the effectiveness of PdO in improving the response time of the In_2_O_3_-based material. Additionally, the incorporation of NiO reduced the recovery time of pristine In_2_O_3_ (or PdO-In_2_O_3_) from the original 232 s (or 674 s) to 168 s (or 336 s).

In a study by Wang et al., it was found that the ethanol gas sensing performance of In_2_O_3_ nanoflowers could be significantly improved by combining them with metal-organic frameworks (MOF)-derived CO_3_O_4_ (Han et al., 2021). The In_2_O_3_ nanoflowers were synthesized *via* a hydrothermal route at 150°C for 10 h. At the optimum operating temperature of 280°C, the CO_3_O_4_-In_2_O_3_ nanoflowers exhibited a sensor response of over 5000 to 100 ppm ethanol. Similarly, the MOFs-derived porous Au@Cr_2_O_3_-In_2_O_3_ nanorods were found to effectively detect 1 ppm isoprene with a sensor response of 6.4 at 180°C (Wu et al., 2022).

#### 2.2.3 In_2_O_3_ composite with other materials

Song et al. reported an outstanding methanol sensing performance of In_2_O_3_ nanocubes composited with Ti_3_C_2_T_x_ MXene at room temperature (Liu et al., 2021b). The In_2_O_3_ nanocubes were synthesized *via* a hydrothermal route at 140°C for 24 h, and the multilayer Ti_3_C_2_T_x_ MXene was synthesized through etching the bulk MAX (Ti_3_AlC_2_) phase with 10 mL HF solution (40 wt%). The In_2_O_3_ nanocubes were modified with a cationic surfactant, (3-aminopropyl) triethoxysilane (APTES), to positively charge their surfaces. The positively charged In_2_O_3_ nanocubes were then mixed with the Ti_3_C_2_T_x_ MXene with negatively charged surfaces, and the mixture was treated under 120°C for a hydrothermal reaction. The In_2_O_3_/Ti_3_C_2_T_x_ composite exhibited typical n-type gas sensing performance to ethanol at room temperature. However, the resistance of the composite after exposure to ethanol was unable to fully recover to its initial level in air, likely because residual methanol was not desorbed from the active site on the surface of the composite at room temperature. The sensor response of the composite to 5 ppm ethanol was ∼29.6 with a response/recovery time of 6.5/3.5 s. The composite also showed a promising gas sensing performance to 5–100 ppm ethanol and was not affected by relative humidity of ∼25–70%. The functional groups of the Ti_3_C_2_T_x_ would be helpful in accelerating the adsorption of ethanol molecules, while the heterojunction between the In_2_O_3_ and the Ti_3_C_2_T_x_ could be another factor improving the ethanol gas sensing performance of the composite.

Song et al. also found that composting In_2_O_3_ nanospheres with Ti_3_C_2_T_x_ MXene nanosheets and Au could improve their HCHO sensing performance (Liu et al., 2022). The Au-In_2_O_3_/Ti_3_C_2_T_x_ composite exhibited a sensor response of approximately 31%, which is higher than that of the pristine Ti_3_C_2_T_x_ (only ∼3.6%). Additionally, the response time and recovery time of the composite were as short as 5 s and 4 s, respectively, to 5 ppm HCHO at room temperature.

Zhu et al. reported an effective enhancement of the H_2_S sensing performance of In_2_O_3_ nanocubes through the use of carbon canohorn (CNH) composites (Zhou et al., 2022). The composite with a CNH mass concentration of 2 wt% (In_2_O_3_/CNH (2 wt%)) exhibited a high sensor response of 2906 to 2 ppm H_2_S at an optimum operating temperature of 70°C. Furthermore, the sensor response of the In_2_O_3_/CNH (2 wt%) to water vapor with 11%–95% humidity was not over 1.52, indicating that humidity did not significantly affect the sensing performance of the composite to H_2_S.

The use of In_2_O_3_-based nanomaterials as gas sensors has been well established, as discussed in the references above. The development of uniform two-dimensional In_2_O_3_ nanomaterials may also lead to surprising gas sensing properties due to their large contact surface with air. Additionally, new materials can be explored to establish novel In_2_O_3_-based composites with heterostructure interfaces, leading to high-performance gas sensors. Careful investigation of the morphology and size effects of the second phase in the composite is essential to screen the best configuration for further improving gas sensing behavior. Machine learning algorithms can be applied to prepare In_2_O_3_-based gas sensors with excellent selectivity, and assembling several gas sensors in a gas sensor array can build a smart gas sensing system capable of simultaneously detecting several gases under mixed gas atmospheres.

## 3 Gas sensing mechanism of In_2_O_3_ nanostructures

**FIGURE 2:**
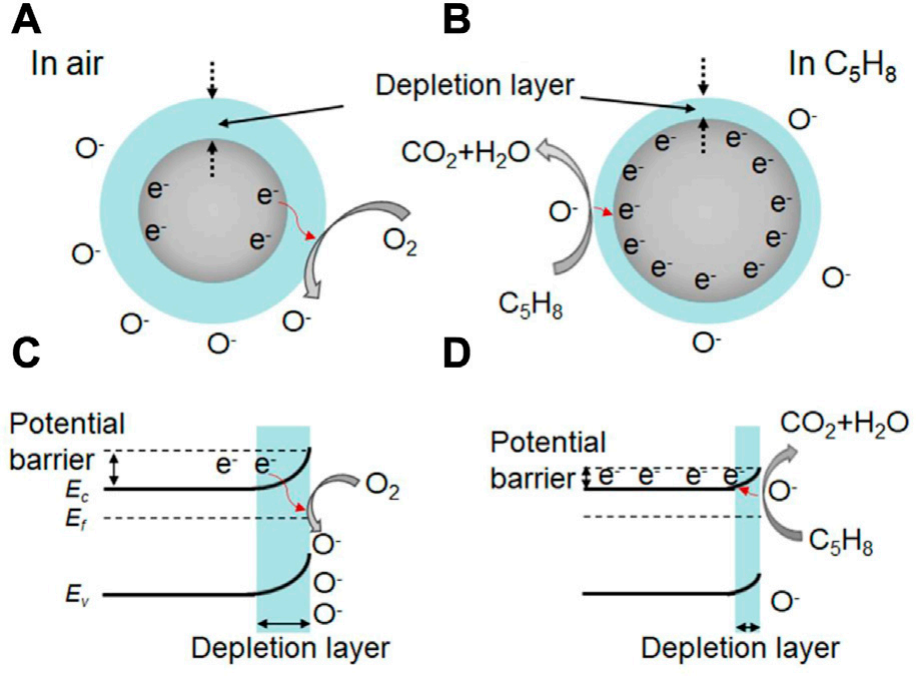
**(A, B)** Schematic diagram of the isoprene sensing mechanism of In_2_O_3_ nanostructure at 190°C, and the corresponding bending of band structures at the surface region of In_2_O_3_ nanosturcture **(C, D)**.

Understanding the gas sensing mechanism is crucial for the development of high-performance gas sensors based on In_2_O_3_. In general, the sensing performance of metal oxide-based sensors is attributed to the redox reaction between the adsorbed oxygen species (O_2_^−^, O^−^ and O^2−^) and the target gas molecules (Wang et al., 2018; Yang et al., 2018; Yang et al., 2019; Wang et al., 2020). For example, flower-like In_2_O_3_ nanostructure have been shown to exhibit a promising sensor response of 3.1 towards 0.5 ppm isoprene at 190°C (Han et al., 2020). In this case, oxygen gas is adsorbed on the active site, forming the adsorbed oxygen molecule in air (Eq. 1). Electrons are then transferred from the conductive bands of the flower-like In_2_O_3_ nanostructure to the adsorbed oxygen molecule, forming adsorbed oxygen species (Eqs. 2–4; Figure 2A). This leads to a bend in the band structure and the formation of a thick space-charge depletion layer at the surface region of the In_2_O_3_ nanostructure (Figure 2C). Moreover, a high potential barrier is created between the contact flower-like In_2_O_3_ nanostructure, resulting in a high resistance in air.

$$O_{2}\;\left(gas\right)\;\to \;O_{2}\;\left(ads\right)$$ (1)

$$O_{2}\;\left(ads\right)+e^{-}\;\to \;O_{2}^{-}\;\left(ads\right)\;\left(T\;<\;100℃\right)$$ (2)

$$O_{2}^{-}\;\left(ads\right)+e^{-}\;\to \;2O^{-}\;\left(ads\right)\;\left(100℃<\;T\;<\;300℃\right)$$ (3)

$$O^{-}\;\left(ads\right)+e^{-}\;\to \;O^{2-}\;\left(ads\right)\;\left(T\;>\;300℃\right)$$ (4)

$$C_{5}H_{8}\;\left(gas\right)\;\to \;C_{5}H_{8}\;\left(ads\right)$$ (5)

$$C_{5}H_{8}\;\left(ads\right)+14O^{-}\;\left(ads\right)=4H_{2}O+5{CO}_{2}+14e^{-}$$ (6)

When the target gas of isoprene is introduced into the testing chamber, the pre-adsorbed oxygen species react with the isoprene molecules (Eqs. 5, 6; Figure 2B), releasing trapped electrons back to the In_2_O_3_ nanostructure. As a result, the thickness of the space-charge depletion layer decreases (Figure 2D), and the potential barrier is also reduced between the contact flower-like In_2_O_3_ nanostructure. This process leads to an effective reduction in the resistance of the sensor and a high sensor response to isoprene Similar theories apply to other In_2_O_3_-based sensors, including Ce-doped In_2_O_3_ microspheres, CeO_2_-loaded In_2_O_3_ hollow spheres, mesoporous In_2_O_3_, or Co-doped In_2_O_3_ nanorods, which exhibit a promising sensing performance to glycol, H_2_, ethanol, or HCHO, respectively (Hu et al., 2018b; Liu et al., 2018; Wang et al., 2018; Cheng et al., 2021).

The high specific surface areas of the In_2_O_3_ nanomaterials have been found to be beneficial in improving the adsorption of gas molecules (Han et al., 2018; Tao et al., 2019; Cao et al., 2020). This increased surface area allows for more gas molecules to access the surface of In_2_O_3_ nanomaterials, promoting the redox reaction between the adsorbed oxygen species and the target gas molecules. Additionally, the formation of a heterojunction between the main phase of In_2_O_3_ and the introduced second phase in the composite can promote the transfer of electrons and holes across their surfaces, leading to the bending of their energy bands and the building of a high potential barrier (Du et al., 2015; Ou et al., 2022). Modulating the height of this potential barrier can dramatically change the resistance of the composite, leading to improved sensing performance (Feng et al., 2015). Overall, these two factors are commonly responsible for the high sensing performance of In_2_O_3_-based composites.

## 4 Conclusion

In this review, we provide a brief overview of current research on gas sensors based on In_2_O_3_ nanostructures. Our analysis shows that uniform In_2_O_3_ nanostructures with high specific surface areas generally exhibit superior gas sensing performance due to enhanced gas molecule adsorption and diffusion. Furthermore, the gas sensing properties of In_2_O_3_-based materials can be effectively enhanced by creating composites. Adding noble metals is a viable strategy for improving the interaction between gas molecules and In_2_O_3_, and metal oxides or Mxenes are widely used to further improve the gas sensing properties of In_2_O_3_ nanostructures. The superior gas sensing performance of composites is primarily attributed to the high specific surface area and the formation of heterojunctions. Therefore, In_2_O_3_-based materials have immense potential for developing gas sensors with exceptional sensing capabilities.
